# Comparison of the Remineralizing Effects of Sodium Fluoride and Bioactive Glass Using Bioerodible Gel Systems

**DOI:** 10.5681/joddd.2009.029

**Published:** 2009-12-15

**Authors:** Attiguppe Ramashetty Prabhakar, Veena Arali

**Affiliations:** ^1^ Professor and Head, Department of Pedodontics and Preventive Dentistry, Bapuji Dental College and Hospital, Davanagere, Karnataka, India; ^2^ Post-graduate Student, Department of Pedodontics and Preventive Dentistry, Bapuji Dental College and Hospital, Davanagere, Karnataka, India

**Keywords:** Bioactive glass, bioerodible gel, drug delivery systems, in vitro caries model, remineralization, sodium fluoride

## Abstract

**Background and aims:**

A carious lesion is the accumulation of numerous episodes of de- and remineralization, rather than a unidirectional demineralization process. Tooth destruction can be arrested or reversed by the frequent delivery of fluoride or calcium/phosphorous ions to the tooth surface. The present study compared and evaluated the remineralization potential of sodium fluoride and bioactive glass delivered through a bioerodible gel system.

**Materials and methods:**

Longitudinal sections of artificial carious lesions, created at the gingivofacial surface of 64 pri-mary maxillary incisors were photographed under a polarized light microscope and quantified for demineralization. The sec-tions were repositioned into the tooth form and randomly mounted in sets of four that simulated an arch form. The teeth were divided into 4 groups: 1) sodium fluoride films, 2) bioactive glass films, 3) control films placed interproximally and 4) non-treatment group. Following exposure to artificial saliva for 30 days, the lesions were again photographed and quantified as above. The recorded values were statistically analyzed using Student’s paired t-test for intragroup comparison, one-way ANOVA and Post-Hoc Tukey’s test for pairwise comparison.

**Results:**

The sodium fluoride and bioactive gel groups showed significant remineralization compared with the control groups (P < 0.001).

**Conclusion:**

Bioerodible gel films can be used to deliver remineralizing agents to enhance remineralization.

## Introduction

Dental research over the last century has advanced our understanding of the etiology and pathogenesis of carious lesions. Increasing knowledge of the dynamic demineralization/remineralization processes has led to the current consensus scientific opinion that a constant supply of low levels of intra-oral fluoride, particularly at the plaque/saliva/enamel interface, is of most benefit in preventing dental caries. Thus, frequent applications of topical fluorides are advised in order to maximize the effectiveness of preventive regimens.^1^

However, most clinical fluoride administration achieves only limited long-term bioavailability. Therefore, slow release devices seem to be an interesting method to sustain low but prolonged fluoride concentrations in saliva.^2^ This approach can be effective in targeting high caries risk groups of the population. Recent clinical studies indicate that slow release fluoride systems are promising cariostatic agents.^1 , 2^

Although fluoride still remains the cornerstone of modern non-invasive dental caries management, new and emerging methods, which can be used as alternatives to fluoride, have been and are in the process of being developed.^2^

Recently, bioactive glass materials have been introduced into many fields of dentistry. This unique material has numerous novel features; the most important feature is its ability to act as a biomimetic mineralizer, matching the body’s own mineralizing traits. Bioactive glass was considered a breakthrough advance in remineralization technology.^3^ Bioactive glass (BAG) consists of minerals that naturally occur in body fluids. It reacts when it comes in contact with water, saliva or other body fluids. This reaction releases calcium, phosphorus, sodium and silicon ions in a way that results in the formation of hydroxyapatite crystals (HAP).^4^ The aim of the present study was to evaluate the effect of slow release of bioactive glass on remineralization of carious lesions as compared to fluoride, when a bioerodible gel system was used.

## Materials and methods

In this study, 64 primary maxillary incisors extracted for therapeutic reasons were included. All teeth were evaluated under a stereomicroscope (Leica Wild M3Z, Germany), using the following inclusion criteria:

- Absence of caries
- Absence of developmental defects
- Absence of enamel fractures and micro-fractures
- Absence of discoloration
- Absence of internal/external pathologic resorption
- No attempted pulp therapy treatment.

All soft tissue and calculus were removed from the teeth with a periodontal scaler. The teeth were cleaned using slurry of pumice and stored in distilled water at room temperature until further use.

A rectangular window measuring 1 mm length by 5 mm width (mesio-distal) was made on the labial cervical third surface of each of the 64 primary maxillary incisor teeth and the remaining portion of each tooth was painted with nail varnish. The window dimensions were confirmed in all the samples, with a Williams periodontal probe to ensure uniformity of the enamel window.^5^

Artificial caries-like lesions were created on the exposed enamel by suspending all teeth in an artificial caries system for 2 days (50 ml per sample).^6^The caries solution consisted of 2.2 mM Ca ^2+^ , 2.2 mM PO _4_^ 3- ^,and 50 mM acetic acid at a pH of 4.4. The solution was kept at a temperature of 37°C, under constant circulation. The solution was changed every 12 hrs.

After two days, the teeth were removed from the artificial caries system. A tooth section of 100 μm thickness (TS 100) was obtained by cutting through the center of the enamel window using a Silverstone-Taylor hard tissue microtome (Tempo instruments and equipments Pvt Ltd, Mumbai, India). These sections were evaluated and photographed under a polarized light microscope (Leica Wild M3Z, Germany). The demineralized areas were then quantified with an imaging system (Image Pro-Plus, Measure and Classify, Media Cybernatics, Inc., Bethesda, MO, USA).

### Quantification of the lesions using Image Pro-Plus

The artificial lesion was quantified at 3 points. The lesion depth was measured from the surface of the lesion to the depth of the lesion, at D1, D2 and D3.^7^ Lesion depth for each section (in μm) was taken as the average of the three representative measurements from the surface of the lesion to the depth of the lesion.

### Preparation of bioerodible gel film

The bioerodible gel films were prepared using polymer/polymer blends along with the drug and a suitable solvent, by casting method. Water soluble polymers—ethylcellulose, HPMC 47CPS, Eudragid RL-100, and Eudragid RS-100—were used for this purpose.^8^ The polymers were weighed accurately and dissolved in their respective solvents. Sodium fluoride (NaF) and Bioactive glass (BAG) were weighed (15 mg) and dissolved in 3 ml of ethanol. The drug solution was then added to the polymer dispersion. A glass mould of size 5×3 cm^2^ was placed on a flat surface and the drug-polymer mixture was poured into it. The mould was kept in hot air oven for 1 hr at 50°C for drying. After this period, an inverted funnel was placed over the mould, overnight to remove the remaining solvent. The film was removed from the mould and placed in wax paper and stored in a dessicator.

### Preparation of samples for remineralization

After quantification, the mounted sections were positioned into the teeth from which they were originally obtained, with a dentin-bonding agent (Prime & Bond NT, Dentsply, Germany) applied only on the outer enamel surface at the incisal edge. Before reassembling the sections, nail varnish was painted on the cut surface of the tooth sections, except for the enamel window. The teeth were then randomly mounted in acrylic blocks in groups of four. The four teeth were arranged to resemble a properly contoured arch form, which were referred to as simulated arch blocks (SAB). The 16 blocks, with four teeth in each block, were divided randomly into control and experimental groups and were color coded as follows:

###  Control groups

Group I (Negative Control, n = 3) included SABs in which 0.5 mm of plain bioerodible gel film was placed interproximally. The SABs were color-coded using yellow colored adhesive tape (3 blocks, 12 teeth).

Group II (Positive Control; n = 3) included SABs that were suspended in artificial saliva. The SABs were color-coded using blue colored adhesive tape (3 blocks, 12 teeth).

### Experimental groups

Group III (n = 5) included SABs in which 0.5 mm of bioerodible gel film impregnated with Sodium Fluoride (NaF) was placed interproximally. The SABs were color-coded using green adhesive tape (5 blocks, 20 teeth).

Group IV (n = 5) included SABs in which 0.5 mm of bioerodible gel film impregnated with BAG (Novamin^ ® ^4505, Alachua, Florida, USA; particle size 5 μm), was placed interproximally. The SABs were color-coded using red adhesive tape (5 blocks, 20 teeth).

Each of the SABs were placed individually in a closed environment with 200 ml media of artificial saliva,^5^ consisting of 20 mM NaH_2_CO_3_, 3 mM NaH_2_PO_4_, and 1 mM CaCl_2_ at 37°C, pH 7, for a period of 30 days with constant circulation. The saliva was changed every 48 hrs.

At the end of the 30-day experimental period, the bonded sections were disassembled. The TS 100, which were used earlier for evaluating the demineralization, were retrieved from each tooth and were oriented longitudinally on the glass cover slides for evaluation under the polarized microscope. The remineralized lesions in all of the four groups were again quantified using an imaging system, the Image Pro-Plus, as described for demineralization.

### Statistical analysis

Results were expressed as mean ± standard deviation, range and percentage changes. Paired t-test was performed to analyze the changes in the depth of demineralization and remineralization. One-way ANOVA was used for multiple group comparison followed by Post-Hoc Tukey’s test for pair-wise comparisons. For all the tests, a p-value of 0.05 or less was considered statistically significant.

## Results

The difference in the mean depth of de- and re-mineralization in the experimental and control groups was statistically significant (P < 0.001). The intra-group comparison of the percentage of remineralization was found to be highly statistically significant in all the groups (P < 0.001) (Table 1). The intergroup comparison of the remineralization potential was statistically significant for all groups except for group I and group II (P = 0.15) and group III and group IV (P = 0.99) (Table 2).

**Table 1:** Comparison of the depth of demineralization and remineralization among the various control and experimental groups

Groups	Demineralization (µm)	Remineralization (µm)	Difference^*^	% of remineralization^†^	t^*^	P
Negative control group						
Group I	71.2 ± 12.0	14.2 ± 3.6	57.0 ± 9.0	21.1 ± 3.3	22.0	< 0.001
Positive control groups						
Group II	68.6 ± 12.0	21.1 ± 5.2	47.5 ± 13.0	30.7 ± 2.5	12.7	< 0.001
Experimental groups						
Group III	69.3 ± 14.8	46.7 ± 10.9	22 .6 ± 4.9	67.7 ± 3.8	20.6	< 0.001
Group IV	64.3 ± 10.4	46.7 ± 7.1	16.6 ± 4.6	73.0 ± 3.0	12.3	< 0.001

^* ^Intra-group comparison by paired t-test; P < 0.001 ^† ^One-way ANOVA; F = 38.2, P < 0.001; Highly significant

**Table 2:** The inter-group comparison of remineralization among the various control and experimental groups

Groups	Mean difference in remineralization (%)	P value of difference in remineralization
I vs. II	6.9	0.44, NS^*^
I vs. III	32.5	< 0.01, S^†^
I vs. IV	32.4	< 0.01, S
II vs. III	25.6	< 0.01, S
II vs. IV	25.5	< 0.01, S
III vs. IV	0.1	0.88, NS

Post-hoc Tukey’s test; Group I: negative control group; group II: positive control group; group III: experimental group (NaF); group IV: experimental group (bioactive glass) ^* ^NS: Not significant, P > 0.05 ^† ^S: Significant P < 0.01, P < 0.05

## Discussion

The mechanisms of fluoride action in the prevention of primary caries are posteruptive through topical effects that can interfere with the dynamic equilibrium at the interface between the mineral surface and oral fluid. The frequent delivery of fluoride to the tooth surface is currently the most efficient measure for the arrestment and reversal of caries; thus, fluoride delivery has caused a paradigm shift where it is now established that incipient non-cavitated enamel caries can be healed.^9^

In our study, the experimental groups included fluoride and bioactive glass, which were delivered through a bioerodible gel system that released fluoride and calcium ions for a longer duration of time.

The greatest degree of depth of remineralization was found in the NaF group, which was similar to the observations made by Sawyer & Donly,^5^ who reported that slow release of fluoride over a prolonged period of time enhanced remineralization.

In a similar study conducted by Mazzavi et al,^10^ who evaluated the efficacy of a bioerodible fluoridated resin on the inhibition of demineralization and compared it with sodium fluoride varnish, found that bioerodible resin group demonstrated less demineralization compared with the sodium fluoride varnish group. They also concluded that the bioerodible resin used in their study was a potential alternative to topical fluoride in the prevention of caries.

An important finding in the remineralization concept was the effect of the concentration of calcium ions on the remineralization process. Over the past several decades, remineralizing or calcifying fluids with variable formulations of calcium, phosphate and fluoride have been developed and investigated in the laboratory.^11^

The BAG group in our study also showed a significant amount of remineralization. The remineralization observed in the BAG group could be attributed to the sustained and prolonged release of ions, which included Ca ^+^ and P ^-^ ions, thus increasing the concentration of available calcium and phosphorus for the remineralization of artificially-created carious lesions.^1^ Bioactive glass in an aqueous environment immediately begins the surface reaction in three phases, leaching and exchange of cations, network dissolution of SiO_2_, and precipitation of calcium and phosphate to form an apatite layer.^13^

Although BAG, with an average particle size of 5 μm, produced a significant amount of remineralization, the percentage remineralization was not significant and was less than that observed with sodium fluoride. Nanometric BAG (45S5) with a particle size of 30 ± 7.8 nm, which was used in a recent study,^14^ was reported to show greater remineralization potential due to the increased surface area, the increased release of ions and Ca/P precipitation in its environment. This implies that the smaller particle size of the bioactive glass enhances the remineralization due to increased surface area.

Another similar study evaluating the remineralization potential of bioactive glass on artificially carious enamel and dentin using Raman Spectroscopy indicated that bioactive glass has the potential for remineralizing artificially carious enamel and dentin.^3^

The positive and the negative control groups also demonstrated a remineralization effect, although at a significantly lower level than the experimental groups, which was similar to previous other studies.^5^ The remineralization was expected to occur as the teeth were placed in artificial saliva, which was supersaturated with calcium and phosphate.

Within the limitations of our study and among the various materials evaluated, it appears that sodium fluoride and BAG, when delivered through a bioerodible gel system, can be used for remineralizing artificial carious lesions. Before extrapolating the results of our study into clinical practice, it must be reiterated that our results were obtained in an ideal in vitro environment free of any microbes. It is, therefore, premature to draw any conclusions regarding the remineralization potential of medicaments delivered through a bioerodible gel system in situ. Saliva, plaque and many other confounding factors may affect the release of remineralizing agents through such systems. We therefore recommend further controlled studies on in vivo models to confirm our observations and ascertain the true clinical efficacy of these newer drug delivery systems.

## Conclusion

The following conclusions were drawn from the present study:

1. Bioactive glass can be used as an alternative to fluoride as a remineralizing agent.
2. Bioactive glass can be delivered using controlled drug delivery systems so that the concentration of calcium and phosphorous can be increased at the target site (carious lesions) so as to enhance the remineralization in the absence of fluoride.
3. The film had the added advantage of being placed in close proximity to the lesions and the method of application was simple.

## References

[1] Toumba KJ (2001). Slow-release devices for fluoride delivery to high-risk individuals. Caries Res.

[2] Bottenberg P, Bultmann C, Graber HG (1998). Distribution of fluoride in the oral cavity after application of a bioadhesive fluoride-releasing tablet. J Dent Res.

[3] Hassanein OE, El-Brolossy TA (2006). An investigation about the remineralization potential of bio-active glass on artificially carious enamel and dentin using Raman spectroscopy. Egypt J Solids.

[4] Oguntebi B, Clark A, Wilson J (1993). Pulp capping with Bioglass and autologous demineralized dentin in miniature swine. J Dent Res.

[5] Sawyer KK, Donly JK (2004). Remineralization effects of a sodium fluoride bioerodible gel. Am J Dent.

[6] ten-Cate JM, Duijsters PP (1982). Alternating demineralization and remineralization of artificial enamel lesions. Caries Res.

[7] Todd MA, Staley RN, Kanellis MJ, Donly KJ, Wefel JS (1999). Effect of a fluoride varnish on demineralization adjacent to orthodontic brackets. Am J Orthod Dentofacial Orthop.

[8] Hillery MA (2001). Advanced drug delivery and targeting: an introduction. In: Hillery MA, Lloyd WA, Swarbrick J, eds. Drug Delivery and Targeting for Pharmacists and Pharmaceutical Scientists.

[9] Aoba T (2004). Solubility properties of human tooth mineral and pathogenesis of dental caries. Oral Dis.

[10] Mazzawi JM, Henson T, Donly KJ (2007). Inhibition of enamel demineralization by a bioerodible fluoridated resin. Am J Dent.

[11] Hicks J, Garcia-Godoy F, Flaitz C (2004). Biological factors in dental caries: role of remineralization and fluoride in the dynamic process of demineralization and remineralization (part3). Clin Pediatr Dent.

[12] Alauddin SS. In vitro remineralization of human enamel with bioactive glass containing dentifrice using confocal microscopy and nanoindentation analysis for early caries defense. [Masters thesis]. Florida: University of Florida; 2004. Available from: http://etd.fcla.edu/UF/UFE0007162/alauddin_s.pdf

[13] Cerruti MM. Characterization of bioactive glasses. Effect of the immersion in solutions that simulate body fluids. [Ph.D thesis]: University of Turin; 2001/2004. Available from: http://altair.physics.ncsu.edu/lkwagner/marta/PhDthesis.pdf

[14] Waltimo T, Brunner TJ, Vollenweider M, Stark WJ, Zehnder M (2007). Antimicrobial effect of nanometric bioactive glass 45S5. J Dent Res.

